# Team Reasoning and the Rational Choice of Payoff-Dominant Outcomes in Games

**DOI:** 10.1007/s11245-018-9575-z

**Published:** 2018-07-10

**Authors:** Natalie Gold, Andrew M. Colman

**Affiliations:** 1 Faculty of Philosophy, University of Oxford, Radcliffe Observatory Quarter, 555 Woodstock Road, Oxford, OX2 6GG UK; 2 School of Psychology, University of Leicester, Leicester, LE1 7RH UK

**Keywords:** Collective rationality, Common knowledge, Cooperation, Common interest game, Coordination, Group identification, Hi-Lo game, Payoff dominance, Prisoner’s dilemma, Stag Hunt game, Team reasoning

## Abstract

Standard game theory cannot explain the selection of payoff-dominant outcomes that are best for all players in common-interest games. Theories of team reasoning can explain why such mutualistic cooperation is rational. They propose that teams can be agents and that individuals in teams can adopt a distinctive mode of reasoning that enables them to do their part in achieving Pareto-dominant outcomes. We show that it can be rational to play payoff-dominant outcomes, given that an agent group identifies. We compare team reasoning to other theories that have been proposed to explain how people can achieve payoff-dominant outcomes, especially with respect to rationality. Some authors have hoped that it would be possible to develop an argument that it is rational to group identify. We identify some large—probably insuperable—problems with this project and sketch some more promising approaches, whereby the normativity of group identification rests on morality.

## Introduction

Game theory is used to explain people’s choices, it both predicts behaviour in strategic situations and provides normative standards for rational play. However, it fails completely in certain elementary cases. It is unable to explain strategic coordination and forms of collective rationality that are familiar features of human strategic interaction. In particular, standard game theory seems powerless to explain the phenomenon where people choose *payoff-dominant* outcomes in games. An outcome is payoff dominant if all players receive a higher payoff than in any other outcome. Nash equilibrium is the central solution concept for non-cooperative game theory and all that it can predict in a game with multiple equilibria is that one of the equilibria should be chosen. If there are outcomes that are Nash equilibria but not payoff dominant, then game theory cannot explain why the payoff-dominant outcome would be played. Further, an outcome is Pareto-dominated if there is another outcome that makes at least one player better off and where no players are worse off. The Nash Equilibrium of a game may be Pareto-dominated by another outcome, but if that outcome is not a Nash equilibrium, then game theory actually predicts that it will *not* be played.

There is a vast literature on cooperation, most of it focused on the Prisoner’s Dilemma, where unilateral defection yields a higher payoff than joint cooperation, so the Nash equilibrium is Pareto-dominated. An interesting and arguably more basic problem is how players coordinate on mutually beneficial equilibria in common interest games, where there is a unique outcome whose payoffs strictly Pareto dominate all other outcomes in the game (Aumann and Sorin 1989). Coordination on the payoff-dominant outcome is clearly a form of cooperation. In Thomas et al. (2014), it is called *mutualistic cooperation* because all the individuals involved in the interaction benefit immediately, as opposed to the *altruistic cooperation* found in games like the prisoner’s dilemma, where cooperation brings benefits to others but at an individual cost. Intuitively, it seems obvious that mutualistic cooperation to achieve the Pareto dominant outcome should be the rational choice in common interest games. However, a principle of rational mutualistic cooperation cannot be derived from the assumptions of standard game theory.

In experimental games and in real life, people often choose payoff-dominant outcomes. If we are prepared to relax the standard assumption of perfect rationality, that players maximize their utilities given their beliefs, which are correct in equilibrium, or the assumption that the players have common knowledge of each other’s rationality, then it is not particularly difficult to construct theories that can explain the choice of payoff-dominant outcomes. However, there are simple coordination games where it seems obvious that players should achieve the payoff-dominant outcomes. It seems strange that we can explain these only by relaxing the assumptions of rationality.

We show how team reasoning, a generalization of game theory with the standard theory as a special case, can explain rational coordination on Pareto-dominant outcomes. Team reasoning applies game theory more widely—it allows that groups can be agents—but it leaves the rationality assumptions of the standard theory intact. We compare the rationality of team reasoning to that of other theories that purport to explain mutualistic cooperation and we explore the type of argument that would be needed to recommend group identification.

## Payoff Dominance in Common Interest Games

**Fig. 1:**
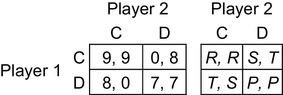
Left: Aumann’s Stag Hunt game, with *R* > *T* > *P* > *S*. Right: generalized template for symmetric 2 × 2 games

In their work on equilibrium selection in noncooperative games, Harsanyi and Selten (1988, pp. 80–90, 355–359) discussed payoff dominance at length, especially in relation to Aumann’s (1987, p. 3) version of the Stag Hunt game, shown on the left in Fig. 1. The game is named after a hypothetical strategic interaction suggested by Rousseau (1755, Part 2, paragraph 9) during a discussion of the early development of civil society. Rousseau imagined hunters who need to coordinate their actions (to choose C) to catch a stag, an endeavour that requires working together, but each is tempted to defect from the joint enterprise (to choose D) and go after a hare, a smaller prize that each could catch without the other’s help. In Aumann’s version of the game, the payoff for joint defection (7) is slightly less than the payoff for unilateral defection (8)—we might imagine that a hunter is slightly less likely to catch a hare if both try the defecting strategy simultaneously, perhaps because both may chase after the same hare—but that in turn is less than the payoff for joint cooperation (9); and unilateral cooperation yields nothing (0).

On the right of Fig. 1 is a template commonly used to define any symmetric 2 × 2 game, according to which Aumann’s Stag Hunt game (1987, p. 3, 1990) is defined by the inequalities *R* > *T* > *P* > *S*. The version originally introduced into game theory and named by Lewis (1969, p. 7) implied the payoff structure *R* > *T* = *P* > *S*, but the strategic properties of Aumann’s and Lewis’s versions are very similar.

In the Stag Hunt game (Fig. 1), the outcome (C, C) is strictly payoff dominant. It is also a *Nash equilibrium* by virtue of the fact that the C strategies are *best replies* to each other. Neither player could get a better payoff by choosing differently against a co-player’s choice of C—against a C-chooser, a player receives 9 by choosing C but only 8 by choosing D—and it follows that neither player has a reason to regret choosing C if the co-player chooses it too. In any game, if there is a uniquely rational profile of strategies (one for each player), this must necessarily be a Nash equilibrium. To see why this is so, we can draw on an important *indirect argument* set out by von Neumann and Morgenstern (1944, Sect. 17.3.3, p. 148).^1^ The assumption of common knowledge of rationality implies that, in any uniquely rational strategy profile, each player will be able to anticipate the co-player’s strategy; but if the strategy profile is not a Nash equilibrium, then at least one player is choosing a strategy that is not a best reply, violating the rationality assumption; therefore the strategy profile must be a Nash equilibrium.

In Aumann’s Stag Hunt game (Fig. 1) a complication arises from the fact that the outcome (D, D) is also a Nash equilibrium—against a D-chooser, a player receives 7 by choosing D but zero by choosing C. The asymmetric (C, D) and (D, C) outcomes are not Nash equilibria. It is tempting to think that rational players, who by definition seek to maximize their own payoffs, will coordinate by choosing C in this game, because the (C, C) equilibrium is better for both than the (D, D) equilibrium. However, remarkably, the fact that (C, C) is (strongly) payoff-dominant does not provide the players with a sufficient reason, derivable from the standard assumptions, to choose their C strategies. The problem is that C is not an unconditionally best choice: it is best only if the co-player chooses C. In fact, against a D-chooser, the best reply is clearly D and not C. In other words, it is rational for a player to choose C if and only if there is a reason to expect the co-player to choose it, so the crucial question is whether a player has sufficient reason to expect a co-player to choose C. There is clearly no such reason, because the game is perfectly symmetric, and the co-player faces exactly the same dilemma. Standard game theory has no way to distinguish between Nash equilibria, or to direct rational players towards more attractive solutions of the game.

The “refinement programme” of standard game theory sought stronger solution concepts than Nash equilibrium, in order to shrink the number of rational solutions in games with multiple Nash equilibria. However, a large number of refinements have been suggested and there are no accepted principles for choosing between them (Kreps 1990). Further, some refinements of game theory suggest that players should choose to hunt hare in the Stag Hunt game. Although the (C, C) equilibrium is payoff dominant, the (D, D) equilibrium is *risk dominant* in a sense defined mathematically by Harsanyi and Selten (1988, pp. 82–89), and risk dominance provides a reason for choosing D. Intuitively, it is obvious that D is much safer: a C choice risks a possible payoff of zero, whereas the worst possible payoff from a D choice is 7.

**Fig. 2:**
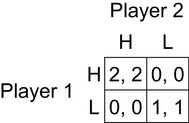
Hi-Lo game, with *R* > *P* > *S* = *T* = 0

The Hi-Lo game shown in Fig. 2 strips the payoff-dominance phenomenon bare and exposes the problem more starkly. Schelling (1960) introduced this game, calling it a “pure common-interest game” (p. 291), and Bacharach named it “Hi-Lo” in unpublished manuscripts and talks in the mid-1990s; the name probably first appeared in print in Bacharach and Stahl (2000). Using the template in Fig. 1 (right), the Hi-Lo game is defined by the inequalities and equalities *R* > *P* > *S* = *T* = 0. It is essentially a Stag Hunt game with the payoffs in the off-diagonal cells set to zero; (H, H) and (L, L) are Nash equilibria, and (H, H) is strongly payoff-dominant over (L, L), as in the Stag Hunt game. Any 2 × 2 game that has positive payoffs (*R, R*) and (*P, P*) in the main diagonal and zero payoffs elsewhere is a Hi-Lo game, provided that *R* > *P* > 0.

In the Hi-Lo game, there is no complication arising from risk dominance, and H seems the obviously rational strategy choice. But, again, each player has a reason to choose H if and only if there is a reason to expect the co-player to choose it also, and there can be no such reason, because the co-player has a reason to choose H if and only if there is a reason to expect the first player to choose it. We are stuck in a vicious circle that provides neither player with any reason, based on the standard assumptions of game theory, to prefer H to L.

In spite of this vicious circle, the Hi-Lo game induces a powerful intuition in human decision makers that H is the rational choice, and experimental evidence confirms that virtually all players choose it (Bardsley et al. 2010). Standard game theory cannot account for this phenomenon of coordination through payoff dominance.

## Some Unsatisfactory Explanations of Mutualistic Cooperation

There are a number of well-known suggestions, with a long pedigree in the literature, that attempt to explain why rational players would achieve payoff dominant outcomes in common interest games (some formulated with respect to Stag Hunt and others with respect to Hi-Lo). However, they are all generally agreed to be flawed.

### Payoff-Dominance Principle

Harsanyi and Selten (1988, pp. 357–359) proposed adding another axiom to the standard rationality assumptions, the *payoff-dominance principle*. According to this principle, rational players choose payoff-dominant equilibria whenever they exist. However, this provides no insight into the phenomenon it is designed to accommodate, and Harsanyi and Selten acknowledged it to be an unsatisfactory and temporary workaround.

Part of the attraction of the idea that a rational solution to a game must be a Nash equilibrium is that Nash equilibria are “self-enforcing”: since every player is doing the best they can, given the strategies of the other players, no-one has any incentive to deviate. Harsanyi and Selten (1988) argued that risk dominance is important only where there is uncertainty about what the other players will do. When there is a single payoff-dominant outcome, it is obvious what the players should do. Therefore one might think that a non-binding agreement to play a payoff dominant equilibrium would be self-enforcing and, hence, a rational solution to the game.

Aumann (1990) argued that non-binding agreements to play the payoff-dominant equilibrium will not always be self-enforcing. If each player has strict preferences over the other’s strategy choice, so she always wants her opponent to choose a particular strategy regardless of what she herself intends to do, then pre-play messages convey information only about what the players want their opponents to do. This information is already common knowledge (because the payoffs of the game are common knowledge). Therefore the pre-play communication conveys no new information about what the player herself intends and, hence, it has no effect on the outcome of the game. In the Stag Hunt, each player strictly prefers that the other plays C (and both players already know this, since the payoffs are common knowledge). Without enforceable agreements, pre-play communication about playing C conveys no information about behaviour and hence does not affect the outcome of the game.

Harsanyi (1995, p. 94, fn. 3) abandoned the payoff-dominance principle, having been convinced by Aumann’s (1990) argument.

### Salience

A *salient* item in a group is “one that stands out from the rest by its uniqueness in some conspicuous respect” (Lewis 1969). Many decades ago, Schelling (1960) showed that people are remarkably adept at using salient *focal points* to solve problems of coordination. There is no doubt that the (H, H) outcome in the Hi-Lo game is a focal point by virtue of the fact that it conspicuously offers higher payoffs than any other outcome.

However, while salience might be part of a psychological explanation of coordination, it is not enough to ground a rational H-choice. Salience singles out a combination of actions as being conspicuous. In order for it to give players a reason to play H, it must single out a combination of actions as being the one that the player should do her part in. So we must ask why the conspicuous combination is the one in which the agent personally has reason to do her part. In answering why the agent has reason to do her part, we cannot appeal to salience, as that would be circular (Gilbert 1989).

Any attempt to derive, from the standard assumptions, a reason for choosing a strategy associated with the payoff-dominant equilibrium generates a version of this vicious circle. This is now generally acknowledged by game theorists (e.g., Anderlini 1999; Aumann and Sorin 1989; Bacharach 2006, Chap. 1; Bardsley et al. 2010; Janssen 2001, 2006).

### Principle of Indifference

The principle of indifference (also called the principle of insufficient reason) is that, if there is no evidence favouring one possibility over another, then we should assign them the same probability. As applied to the Hi-Lo game, the principle of indifference suggests that, since Player 1 (for example) has no reason to expect Player 2 to choose H or L in the Hi-Lo game, then Player 1 should assign equal probabilities to each of these choices. Under that assumption, Player 1’s expected payoff from choosing H is obviously higher than from choosing L, because ½(2) + ½(0) = 1 and ½(0) + ½(1) = ½, therefore Player 1 will choose the expected-payoff-maximizing strategy H.

This argument is fallacious because the problem is not one of individual decision making, where standard decision theory and simple expected utility maximization apply: Player 2 is not indifferent Nature but an intelligent player who can and will formulate and respond to expectations about Player 1’s intentions. If the argument from the principle of indifference were valid, then by the common knowledge assumption, Player 2 would anticipate Player 1’s choice of H and would choose a best reply to it, namely H. But this means that Player 2 would choose H *with certainty*, and that contradicts the assumption on which Player 1’s argument for choosing H is based, namely that Player 2 is equally likely to choose H or L.

Closely related fallacies based on probabilities are discussed in greater depth by Colman et al. (2014).

## Team Reasoning Solves Common Interest Games

According to theories of team reasoning, players solve common-interest games like Hi-Lo or Stag Hunt by adopting a distinctive mode of reasoning from preferences to strategy choices (Bacharach 1999, 2006; Sugden 1993, 2003, 2015). Standard game-theoretic reasoning amounts to asking *What do I want?* and, given my knowledge of the game and my expectations of what my co-player(s) will do, *What should I do to achieve this?* Team reasoning alters the unit of agency from the individual to the pair or more generally the group of players by allowing each player to ask *What do we want?* and *What should I do to play my part in achieving this?* Team-reasoning players first search for an outcome that would be best for the group of players; if such an outcome exists and is unique, then they identify and play their component strategies of the jointly optimal strategy profile.^2^ Within this theoretical framework, standard individual reasoning is merely a special case of team reasoning when the team has only one member (Gold and Sugden 2007a, b).

Team reasoning provides a solution to the problems of coordination and payoff dominance as follows. In Aumann’s Stag Hunt game (Fig. 1), a team-reasoning player notes that the (C, C) strategy profile is uniquely optimal for the player pair, because it offers the best possible payoff to both, and no other strategy profile yields either player a payoff as good as the payoff in (C, C). If there is common knowledge that both players adopt the team-reasoning mode of choosing their strategies, both therefore select and play their C strategies. It is essentially the same in the Hi-Lo game shown in Fig. 2. The (H, H) strategy profile is uniquely optimal for the player pair, because it yields the best possible payoffs to both players, therefore team-reasoning players select and play their H strategies. Team reasoning solves any coordination game with a payoff-dominant outcome in the same way.

Team reasoning involves both a preference transformation and an agency transformation. There is a preference transformation, from the individual to the group (or *team*) preferences. There is also an agency transformation, with the group of players being treated as a single entity. The agency transformation is essential to solving common interest games. To see this, consider the two-player Hi-Lo game. A theory that only involved payoff transformations and not agency transformations would not change the recommendations of standard game theory. Imagine that the players each care about the other player’s outcome and that they apply some weight λ and (1 − λ) to their outcome and the other player’s outcome respectively. In a two player game, if each player *i*’s individual outcome is *x*_*i*_ then we can represent the transformed utility as λ*x*_*i*_ + (1 − λ)*x*_*j*_; λ = 1 corresponds to complete egoism and λ = 0 to complete altruism. (This is a pretty standard approach; Collard 1978, explores its implications for the fundamental theorems of economics). In Hi-Lo, *x*_*i*_ = *x*_*j*_ in all outcomes so, whatever the value of λ, the payoff matrix does not change. Therefore there is still an equilibrium selection problem. The agency transformation part of team reasoning allows profile selection. From the perspective of the team, Hi-Lo has a uniquely best profile, so the theory of team reasoning can predict H-play, provided there is common knowledge of team reasoning. Similarly, the best that can be hoped for with the transformed Stag Hunt game is that it will become a Hi-Lo, where the two players’ interests are completely aligned but there are still two Nash equilibria, but with common knowledge of group identification the theory of team reasoning can predict C-play. In the next section, we discuss what happens if this common knowledge assumption is relaxed.

## Team Reasoning and Pareto-Dominant but Non-equilibrium Outcomes

**Fig. 3:**
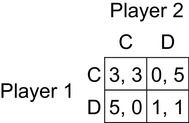
Prisoner’s Dilemma game, with *T* > *R* > *P* > *S* and 2*R* > *S* + *T*

Although team reasoning was originally developed to solve the problem of coordination in Hi-Lo, it also provides a compelling explanation for altruistic cooperation in the Prisoner’s Dilemma. Figure 3 shows the Prisoner’s Dilemma game (*T* > *R* > *P* > *S* and 2*R* > *S* + *T*) with the now conventional payoff values popularized by Axelrod (1984). It has a unique Nash equilibrium at (D, D), and the D strategy is a strongly dominant strategy for both players in the sense that it yields a better payoff than C irrespective of the co-player’s choice. Nevertheless, experimental studies have invariably revealed that many human decision makers cooperate, even when the game is played just once, and multiplayer versions of this game also elicit frequent cooperation (Balliet and Van Lange 2012).

Team reasoning explains cooperation in the prisoner’s dilemma very easily. In Fig. 3, if a team-reasoning player interprets the collective payoff in the simplest and most intuitive way, as simply the sum of payoffs to the two players, then it becomes obvious that the (C, C) strategy profile is uniquely optimal for the player pair because 3 + 3 = 6 is a larger joint payoff than in any other outcome. (See Gold 2012; Gold 2018; Colman and Gold 2017, or Karpus and Gold 2016 for discussion of different interpretations of the payoff function.) If there is common knowledge that both players adopt the team-reasoning mode, then both will play C, their component strategies in this uniquely optimal profile. But what happens if the common knowledge condition is not fulfilled?

Bacharach (1999, 2006) developed a theory of *unreliable team interaction* that allows the possibility of team reasoning when players are uncertain whether or not other players will use team reasoning. Let us say that an individual “identifies” with a group if she conceives of that group as a unit of agency, acting as a single entity in pursuit of some single objective (Gold and Sugden 2007a, b; Gold 2018). For Bacharach, whether a player identifies with a particular group is a matter of psychology, or how she *frames* the decision problem. He defined a frame as the set of concepts that a player uses to conceptualize a problem. In order to engage in team reasoning and ask *What should we do?*, a player’s frame must include the concept “we.” Players who are in such a *we*-frame will adopt team reasoning. (This telescopes several steps in a single process. For critical discussion, see Gold 2012).

Bacharach’s theory includes a parameter ω (0 ≤ ω ≤ 1), representing the probability that any individual player will be in a *we-*frame, and hence group identify and team reason. In Bacharach’s (1999, 2006) presentation, the value of ω is common knowledge among all players. In Gold and Sugden’s (2006, 2007a, b) presentation of Bacharach’s theory, the value of ω is assumed to be common knowledge only among those players who group identify. One reason for making this change stems from Bacharach’s theory of framing. Bacharach often compares framing to seeing. If his idea is that players who reason individualistically do not have the concept “we” in their frame, then it is hard to see how they can even recognise the possibility of the *we-*frame, in which case they cannot have beliefs about the probability that others adopt it and are team reasoners. An alternative way to reconcile framing and knowledge about ω, which would allow individualistic reasoners to have beliefs about ω, would be to have an interpretation of framing that is not only ‘seeing’ but also ‘endorsing’ particular concepts as the lens through which to interpret the situation, and those concepts having ‘motivational grip’ (Gold 2012). The ω is the probability that the *we*-frame has motivational grip. In either case, team reasoners then maximize the expected team payoff, taking into account the probability of group identification by the co-player(s). As in standard game theory, in equilibrium all beliefs turn out to be correct.

In Bacharach’s theory, a player will sometimes adopt the team-reasoning mode even without assurance that the co-player(s) have identified with the group, that is, when ω < 1, and may thus end up receiving a worse individual payoff than expected. His theory also allows the possibility that the off-diagonal outcomes in the prisoner’s dilemma, (C, D) and (D, C), are ranked higher by the team than the Nash equilibrium outcome, (D, D). In this case, a team reasoning player could play C despite a belief that the co-player(s) will certainly reason individually (ω = 0). Assuming once again that the collective payoff is simply the sum of individual payoffs, a simple and clear case in point is the Prisoner’s Dilemma game shown in Fig. 3. In this game, whenever one player cooperates and the other defects, the collective payoff to the player pair (5) is greater than when both defect (2); therefore even if ω = 0, a team-reasoning player will choose C, expecting a personal payoff of zero. This is not an issue for Bacharach, since a team reasoner considers that what matters for her is the team payoff and does what is instrumentally rational for the team; in contrast, it is something that Sugden (1993, 2003, 2015) is deeply concerned about.

In Sugden’s (1993, 2003) theory, a player never deliberately pays a personal cost for team reasoning. For Sugden, individuals are motivated to adopt the team-reasoning mode only by a promise of a better individual outcome for everyone involved in the interaction. Sugden (2011) casts his version of team reasoning as benefiting each individual in comparison to a noncooperative default. Sugden (2015) defines the default as each player’s maximin payoff, the highest payoff that she can guarantee herself independently of other players’ strategy choices, and says that each player must benefit relative to this benchmark. (See Karpus and Radzvilas 2017, for a formalization of this.) It follows that, for Sugden, the collective utility function, or later (Sugden 2015) the goal of achieving common interests, can never make an individual player worse off, and in a Prisoner’s Dilemma, a team will never prefer an asymmetric outcome that gives one player a poor personal payoff to joint defection with better personal payoffs but a worse collective payoff.^3^

For the same reason, in Sugden’s theory a player will not adopt team reasoning without having assurance that the co-player will also cooperate. Players engage in team reasoning only if they have reason to believe that the other player(s) identify with the group, endorse the idea of mutually assured team reasoning, and accept the idea that the goal is to maximize the collective payoff of the group (Sugden 2003), or (in later writings) to achieve the group’s common interests. Reason to believe could be generated, for example, by a tannoy announcement or by induction from a previously existing convention.

In later writings, Sugden (2011, 2015) takes pains to differentiate his version of team reasoning from that of Bacharach (1999, 2006). Sugden (2011) contrasts his version of team reasoning with that of Bacharach, comparing their different interpretations of the question ‘*What is it rational for us do?*’. For Bacharach, this involves maximising a team objective, whereas Sugden (2011) re-interprets the question as ‘*What are the terms of an agreement that it would be rational for each to make if all could be assured that the agreement would be kept*?’. For Sugden, the benchmark for team reasoning is the payoffs that the payers could achieve as individuals and he prefers to speak of the players’ ‘common interests’ instead of a team utility function; he makes a deliberate move to the language of cooperative game theory (Sugden 2015). To some extent, this move is rhetorical. It is still possible to model Sugden’s provisos using expected utility theory (Gold 2012). The need for mutual benefit and the introduction of cooperative game theory can be thought of as putting constraints on the team utility function, the need for assurance suggests that players will not team reason unless ω = 1.

Later Sugden (2011, 2015) says that a convention can be a team reasoning solution even if it is not optimal, so long as it is mutually beneficial compared to individuals’ maximin payoffs. Under this definition, (L, L) can be a team reasoning solution of Hi-Lo. Thus the most recent version of Sugden’s theory no longer possesses the advantage of predicting payoff-dominant outcomes as the unique team reasoning solution and therefore it is out of the scope of this paper.

In Prisoner’s Dilemmas, as in common interest games, agency transformation is required as well as a payoff transformation. This can be revealed by applying the transformation above, where the individual *x*_*i*_’s utility is λ*x*_*i*_ + (1 – λ) *x*_*j*_, to the Prisoner’s Dilemma in Fig. 3. If λ = 1/2, then the game is transformed into a Hi-Lo game (see Gold and Sugden 2007b). In fact a common feature of most “solutions” to the Prisoner’s Dilemma is that they transform (C, C) into an equilibrium but do not change the equilibrium status of (D, D) (Gold 2012). Depending on the values of the off-diagonal payoffs, they either transform the Prisoner’s Dilemma into a Hi-Lo game or a Stag Hunt. As we saw above, to definitely solve these games, team reasoning is needed to de-conditionalize players’ strategies. Even completely self-sacrificing players cannot solve their problem in the Prisoner’s Dilemma. If λ = 1, we have what Parfit (1984) calls “the altruists dilemma”: the players each want to play D in order to benefit the other (and this remains the dominant strategy) but they could have each helped the other more if they had both agreed to play C.

## Other Explanations of Mutualistic Cooperation Assessed with Respect to Their Rationality

Team reasoning is not the only current explanation of mutualistic cooperation. There is empirical evidence to support the idea that people team reason in common interest games (Bardsley and Ule 2017; Bardsley et al. 2010; Butler 2012; Gold and Thom, unpublished manuscript), but there is also empirical evidence to support other theories. We explain the fundamental ideas behind the principle alternative theories in relation to the simple Hi-Lo game shown in Fig. 2 and discuss how they relate to rationality. We do not consider the psychology of mutualistic cooperation. Readers interested in that can consult a companion paper (Colman and Gold 2017).

### Social Projection Theory and Evidential Reasoning

According to social projection theory (Acevedo and Krueger 2005; Krueger 2007; Krueger et al. 2012), most people expect others to behave as they do, and they therefore assume that, if they choose H (Fig. 2), then the co-player is also likely to choose H. It follows that a player expects to receive a payoff of 2 by choosing H and 1 by choosing L, and this provides a reason for choosing H. Al-Nowaihi and Dhami (2015) have recently developed a formal equilibrium concept based on this theory.

Social projection theory provides a psychological but not necessarily a rational mechanism for H-choice. It is related to what economists and philosophers call *evidential reasoning* or *magical thinking*, which is generally regarded as irrational (Binmore 1992; Elster 1989; Joyce 1999; Lewis 1979; Quattrone and Tversky 1984). The problem is that it mistakes correlation for causation. The irrationality of doing this is often discussed with respect to the Prisoner’s Dilemma (Lewis 1979). If there is reason to believe that the two players are similar, then a player who is considering playing D has evidence that the other player is also considering playing D and a player considering playing C has evidence that the other player is considering doing likewise. Nevertheless, the dominant strategy is still to play D, as it gives a higher playoff regardless of what the other player chooses. If the other player is going to choose C, then our reasoner would be better off choosing D. If choosing D is a sign that the other player is also likely to play D, then this is simply bad news. It does not have any causal import.

In economics, modellers have incorporated evidential reasoning by introducing action-dependent beliefs and allowing players to maximize expected utility conditional on their own action (Hammond 2009; Mandler 2007; Masel 2007). In a Bayesian model of belief formation, is possible to rationalise a player having beliefs about other players that depend her action (Board 2006; Larrouy and Lecouteux 2017). Never-the-less, the correlations between the players’ actions are not causal correlations and ignoring the lack of causality is generally believed to be irrational (Lewis 1979; Gibbard and Harper 1978). Rational action requires ‘a proper kind of connection to desires, beliefs, and evidence’ (Elster 1986, p. 2) and correlations that are not causal are not considered to be a proper kind of connection. Indeed, the majority of the economists modelling action-dependent beliefs intend to model a psychological phenomenon and are at best agnostic about its rationality (Hammond 2009; Masel 2007; Larrouy and Lecouteux 2017).

There is an extended debate about the rationality of evidential reasoning, but the majority of decision theorists come down against it. For more discussion see Weirich (2016).

### Cognitive Hierarchy Theory

Cognitive hierarchy and Level-*k* theories (Camerer et al. 2004; Stahl and Wilson 1994, 1995) are designed to model players who reason with varying levels of strategic depth. Level-0 players have no beliefs about their co-players and choose strategies either randomly, with uniform probability, or by using simple heuristics such as salience; Level-1 players maximize their own payoffs relative to a belief that their co-players are Level-0 players; Level-2 players maximize their own payoffs relative to a belief that their co-players are Level-1 players; and so on. Experiments have confirmed the findings of Camerer et al. that Level-1 is most common, followed by Level-2 (Bardsley et al. 2010; Colman et al. 2014), although there is a worry that Level-0 behaviours may not be consistent across games (Heap et al. 2014).

Cognitive hierarchy theory can predict H-choice in Hi-Lo. If a Level-1 player assumes that her co-player is choosing between H and L randomly, with equal probability, then choosing H yields an expected payoff of 1, whereas choosing L yields ½. Hence she concludes that she should choose H. Level-2 players choose H because they expect their co-players to choose H with certainty, and the same applies at higher levels.

This may provide a model of behaviour, but it is not rational behaviour according to standard game theory. The choices of Level-0 players are being treated as parametric, not as the choices of rationally responding players, similar to the criticism of the principle of insufficient reason above. Further, even for those levels that are reasoning, their beliefs about the distribution of other players in the population (the percentage of level-0, 1, and 2 s) will often turn out to be incorrect.

### Strong Stackelberg Reasoning

Strong Stackelberg reasoning (Colman and Bacharach 1997; Colman et al. 2014; Colman and Stirk 1998; Pulford et al. 2014) entails an assumption that players choose strategies as though their co-players could anticipate their choices. Thus, a Stackelberg reasoner chooses as though expecting a choice of H to be anticipated by the co-player, who would therefore choose H, and an L choice to be met with an L choice by the co-player for the same reason. The Stackelberg reasoner gets a better payoff in the first case than the second and therefore chooses H.

Stackelberg reasoning is a generalization of the “minorant” and “majorant” models introduced by von Neumann and Morgenstern (1944), Sect. 14.4.1, pp. 100–104, in their analysis of zero-sum games. von Neumann and Morgenstern’s objective was to determine how a rational player could exploit the payoff structure of the game to get the maximum payoff that can be obtained independently of the rival’s behaviour (Giocoli 2003, p. 265). They introduced the “minorant” and “majorant” games as a technique to identify the range of payoffs that a player could guarantee, if she were in the best or worse possible situation. They made the assumption that players reason as though any strategy choice will be anticipated by the co-player, which is also made in Strong Stackleberg reasoning, and used it to rationalize their solution of zero sum games.^4^ Similiarly, in Strong Stackleberg reasoning, the objective is to determine how a rational player could exploit the payoff structure of the game to get the maximum payoff that can be obtained independently of the rival’s behaviour. In Stackelberg-soluble games, it yields precisely the strategy that maximizes one’s payoff irrespective of the worst that the co-player could do in response (i.e., assuming, exactly as in von Neumann and Morgenstern, that the co-player always does what would be worst from the first player’s viewpoint).

Strong Stackelberg reasoning differs from social projection theory because social projection theory requires that players believe that their co-players’ actions are dependent on their own, but in Stackleberg reasoning they merely behave as though this were the case as a heuristic device to clarify the situation. There is no necessary assumption that people who use strong Stackelberg reasoning actually *believe* that others can anticipate their choices, merely that they act *as though* this were the case, and the player’s beliefs about the other players’ strategy choices turn out to be correct in equilibrium.

### Virtual Bargaining

The theory of virtual bargaining (Misyak and Chater 2014; Misyak et al. 2014) proposes that individuals reason about problems of coordination by considering what strategies they would agree on if they could bargain or negotiate explicitly. In the Hi-Lo game and arguably also in the Stag Hunt game, it is obvious what bargain they would arrive at, hence communication is unnecessary and they choose the appropriate H or C strategies directly.

In terms of rationality, virtual bargaining suffers from a similar problem to that in the discussion of salience, above: Why does the fact that a combination *would be* the outcome of bargaining give the agents a reason to choose it? We can even grant that bargaining and contracts give reasons, but in this case no bargaining has actually taken place. This echoes Dworkin’s (1973, p. 501) line about hypothetical social contract theories, that ‘A hypothetical contract is not simply a pale form of an actual contract; it is no contract at all.’ Dworkin’s point was that there are no grounds to enforce a hypothetical contract; equally there are no rational grounds to expect that other players will do their part.

## The Rationality of Team Reasoning

Instrumental practical reasoning presupposes a unit of agency. Standard game theory limits the unit of agency to the individual. The theory of team reasoning allows that there can be multiple levels of agency and includes them in the model. It is a theory of choices made by group members, who think about what they should do as a member of the group, so the standard theory is a special case when the number of group members equals one (Gold and Sugden 2007a, b). (A similar approach can be used with choice over time, where standard theory adopts the time-slice as the unit of agency and team reasoning can introduce the level of the individual Gold 2013; Gold, in press.) Team reasoning introduces a new primitive into the theory, about the level of agency or the team, and also players’ beliefs about the level of agency (whether other players will group identify), which turn out to be correct in equilibrium. Therefore, the theory of team reasoning can conclude that, when a player identifies with the group, it is rational for her to choose the strategy that leads to the payoff-dominant outcome. Game theory, like expected utility theory, is a theory of instrumental rationality. Its normative recommendations are about how a player can best achieve her ends (given her beliefs), but it takes those ends as given; they are prior to instrumental rationality. The phenomenon of payoff-dominance poses a problem for standard game theory because a unique recommendation to play the strategies involved in payoff-dominant outcomes requires the individual to do what is best for the group. By allowing that groups can be agents, the theory of team reasoning allows that the group can be instrumentally rational, and shows how outcomes that are good for the group can be achieved through the rational decision-making of the individual members, given that they group identify. Therefore, according to the theory of team reasoning, if there is a team agent, then the pay-off dominant outcome of a game is the unique rational solution. It explains mutualistic cooperation while maintaining the same connections as standard game theory between preferences, evidence, and actions.^5^

This leaves the questions of how individuals come to identify with group and whether it can be instrumentally rational for them to do so. Bacharach and Sugden differ on the question of how but agree that group identification is not the subject of rational choice. For Bacharach (2006), group identification is a psychological process and therefore not amenable to rational choice. His hypothesis is that games with a single Pareto-dominant outcome will tend to induce group identification, but this is an empirical hypothesis. In his theory, the evaluation of options is done by an agent who is already using a frame, there is no point where an agent can evaluate which frame would be better to adopt. For Sugden (1993, 2000, 2003), group identification is a matter of choice, but nevertheless the choice cannot be evaluated with respect to instrumental rationality (Gold and Sugden 2007a, b). Sugden thinks that people may choose to team reason in situations where there is the possibility of mutual advantage, but they are not rationally required to. For him, as for Bacharach, all goals are the goals of agents and it is not possible to evaluate those goals without first specifying the unit of agency.

In contrast, Hurley (1989, p. 145) says that “an adequate theory of rational choice should address the question of what the unit of agency among those possible should be”. She suggests that the source of evaluation of outcomes is not given by the unit of agency. There are two ways that the source of evaluation could come apart from the unit of agency and neither of them are promising routes for a theory of instrumentally rational choice of the unit of agency.

A closer look at Hurley’s position reveals the bind. Hurley says that, “As an individual I can recognise that a collective unit of which I am merely a part can bring about outcomes that I prefer to any that I could bring about by acting as an individual unit.” (Hurley 2005, p. 203). The most straightforward reading of this is that we should privilege the individual perspective and individual goals. However, once we acknowledge that there are multiple levels of agency, it is not clear why one should privilege the individual level over the group level for the purpose of making evaluations. Intuitively, individuals are privileged in our ontology; the question is whether instrumental rationality requires privileging individual goals or whether some other form of argument is needed to fill the gap.

In the context of standard game theory and instrumental rationality, one might think that individuals are privileged in virtue of being the units that make choices. However, that is not sufficient to make them the unit of rational evaluations—it is actually not even strictly speaking true, as we can see by considering how rational choice theory models individuals who make a series of choices over time. Here, the individuals is modelled as a sequence of selves at a time, or ‘timeslices’, and different timeslices may have different preferences. This can lead to strategic interactions between timeslices, which we might think of as intra-personal games, and which lead to problems of coordination and cooperation that parallel those in the inter-personal case (Read 2001; Gold 2013; Gold, in press). This way of modelling the self over time is natural because the timeslice is the locus of choice. From this, we can take two lessons. First, even in game theory, the real locus of decision-making is the timeslice, it is just that in most games the individual players’ preferences are stable over the time frame of the interaction, so we can model them as one single individual preference ordering. Second, once we think of individuals like this, the case for privileging the evaluative stance of the locus of decision making looks less appealing (although Parfit 1984 does take that position). That would involve privileging the timeslice in the intra-personal case, whereas most people’s intuition is that it is the individual over time that is privileged, not the timeslice.

People have expressed the hope that principles of rational identification can be found. The most promising place to look for them seems to be in an account of the worth of a life and the importance of life projects. However, this is beginning to look like a project in ethics or morality, not one in rationality, except maybe in a thick Kantian sense of rationality where the moral law is a truth that can be discovered by reason. Even if such a theory can be constructed, it will not be a theory of instrumental rationality. For instance, Parfit (1984) argues that rationality resides with the timeslice, but that the timeslice may be required to do what is best for the individual or the group as a part of a consequentialist morality. In contrast, Korsgaard (1989) argues that the rationality of pursuing intertemporal projects implies that it is rational to prioritise the individual rather than the timeslice. But Korsgaard’s is a Kantian conception of rationality, where it is rational to do what we have reason to do. Therefore, it is a thick conception of rationality, in the sense that it judges the rationality of ends, and not merely a thin instrumental theory of rationality, which takes the agent’s ends as given.

An alternative way of reading Hurley is that we should look for an agent-neutral perspective from which to evaluate the options, while acknowledging that all thinking is done by individuals. It is easy to see how an agent-neutral perspective enters the picture if a moral evaluation is being made. For instance, Utilitarianism is an agent-neutral moral theory; an agent could make a Utilitarian evaluation and then use that to decide what team membership to adopt. Regan (1980) offers such a theory. However, the agents in this theory have already chosen to adopt moral ends. We lack a story about why a rational agent should adopt moral ends. This is an old chestnut, going back at least as far as Plato’s *Republic*, which still lacks a satisfactory solution. Further, it seems that there are insuperable obstacles to solving the problem of rational group identification within a theory of purely instrumental rationality. A reasoning process already presumes an agent who is doing the reasoning. As Bardsley (2001: 185) puts it, “the question should I ask myself “what am I to do?” or “what are we to do?”? presupposes a first person singular point of view”. Therefore it is hard to see how an instrumentally rational process of evaluation of the unit of agency can occur.

A similar problem holds for Gauthier (1986), who has long held that it can be instrumentally rational to cooperate in the Prisoner’s Dilemma game. In a recent re-working of his theory, Gauthier (2013) contrasts two opposed conceptions of deliberative rationality: maximization, which is equivalent to individualistic best-response reasoning, and Pareto-optimization, which is equivalent to team reasoning, although he doesn’t call it that (Karpus and Gold 2016). Gauthier suggests that Pareto-optimization is a necessary condition for rationality in multi-player games. His justification for team reasoning is that it would pass a contractarian test, so Pareto-optimization is, as he puts it, a part of “social morality” (2013, p. 624). His position is very similar to that of Sugden (2015), except that Gauthier holds that social morality is part of instrumentally rational choice. However, Gauthier recognises that he lacks an argument to support this. For the reasons explained above, we are pessimistic that such an argument exists.

Never-the-less, team reasoning is an important addition to game theory because it allows that groups can be agents and shows how individuals who obey the standard rationality assumptions can do their part in achieving the outcome that is instrumentally rational for the group. This is important for economics and decision theory. In decision theory, an agent is an entity with preferences that acts instrumentally to achieve its ends (given its beliefs), usually modelled as maximizing a utility function. In economics, it is actually standard to model some groups as agents, most obviously firms, but also diverse groups such as families, political parties, and trade unions. The theory of team reasoning is important because it makes space for individual and collective rationality in the same model, and shows how pursuing the group objective can be rational for the individual team members, within the standard assumptions of game theoretic rationality. Team reasoning is also relevant for philosophers who want to connect individual and collective action, and individual and collective intentions (Gold and Sugden 2007a, b). However, anyone making an argument that it is normatively required to group identify and team reason will have to draw on resources other than instrumental rationality.

## Conclusion

Although the Prisoner’s Dilemma has garnered most attention, problems of mutualistic cooperation are fundamental. Team reasoning can explain rational coordination on mutualistically cooperative outcomes, such as (C, C) in the Stag Hunt and (H, H) in Hi-Lo. The theory of team reasoning generalizes game theory, by introducing the possibility that groups can be the unit of agency, with standard game theory as a special case when the group only consists of one player. Team reasoning can also explain why it is sometimes rational to cooperate in a Prisoner’s Dilemma, where the Pareto-dominant outcome is not a Nash equilibrium.

Team reasoning explains how it can be rational to cooperate, within the rationality assumptions of standard game theory. Theories of team reasoning allow that groups can be instrumentally rational agents with goals (which we can represent as team preference orderings) and that individuals within groups can rationally choose to do their parts in order to achieve the outcome that is ranked highest by the group. Some people have hoped we can find an argument that it is instrumentally rational to group identify. But any theory of the rational choice of the unit of agency faces the problem of how to specify goals and evaluate outcomes independent of the unit of agency. If we take our goals from our moral theory, that turns team reasoning from a theory of rational choice into a theory of moral choice, which is not intended by many of its proponents. It does not seem that one can choose one’s ultimate goals using a theory of rational choice.

## Footnotes

1 This argument pre-dates Nash’s (1951) presentation of his equilibrium concept for non-cooperative games and was intended as a general principal for identifying rational solutions of zero-sum games.

2 The theory is not well developed in the case where there is no uniquely best outcome, although it seems that some versions of the theory could accommodate this possibility. Bacharach’s (2006) theory is an obvious candidate for this, if we allow the team reasoning solution to admit mixed strategies. This seems plausible, since Bacharach’s theory already allows that group-identifying individuals maximize the expected team utility, which is required when playing mixed strategies. Sugden (2015, p. 156) modifies his original (1991, 1993) position so that it does not require individuals to identify a uniquely team-optimal solution. He says that mutually beneficial conventions can be team reasoning solutions even if players would not be able to discover these conventions by reasoning about optimal solutions.

3 Sugden’s position has changed over the years. Sugden (2000) takes a line very close to Bacharach’s, saying that the team utility function represents the preferences of the group and “It does seem possible in fact for people to construe themselves as members of a plural agent whose collective objective is contrary to the unanimous preferences of those members”. However, he has since moved away from this position.

4 In their strictly competitive (2-player, zero-sum) case, Stackelberg reasoning always converges on an equilibrium, hence all such games are Stackelberg-soluble, whereas in the general case (including pure coordination and mixed-motive games) it doesn’t necessarily converge.

5 This paragraph is true of Bacharch’s theory of team reasoning and is also explicit in Sugden (2000) and Gold & Sugden (2007a, b), although in more recent work Sugden has moved away from the conception of team reasoning as instrumental rationality for groups (Sugden 2015).
